# Supplementary material for: Filtration effect of Cordyceps chanhua mycoderm on bacteria and its transport function on nitrogen
Source: Microbiol Spectr. 2023 Dec 15;12(1):e01179-23. doi: 10.1128/spectrum.01179-23 (PMC10783027; doi:10.1128/spectrum.01179-23)
Supplement: Supplemental material — Fig. S1 to Fig. S9; Tables S1 to S3. [file spectrum.01179-23-s0001.docx]

**Supplementary Material**


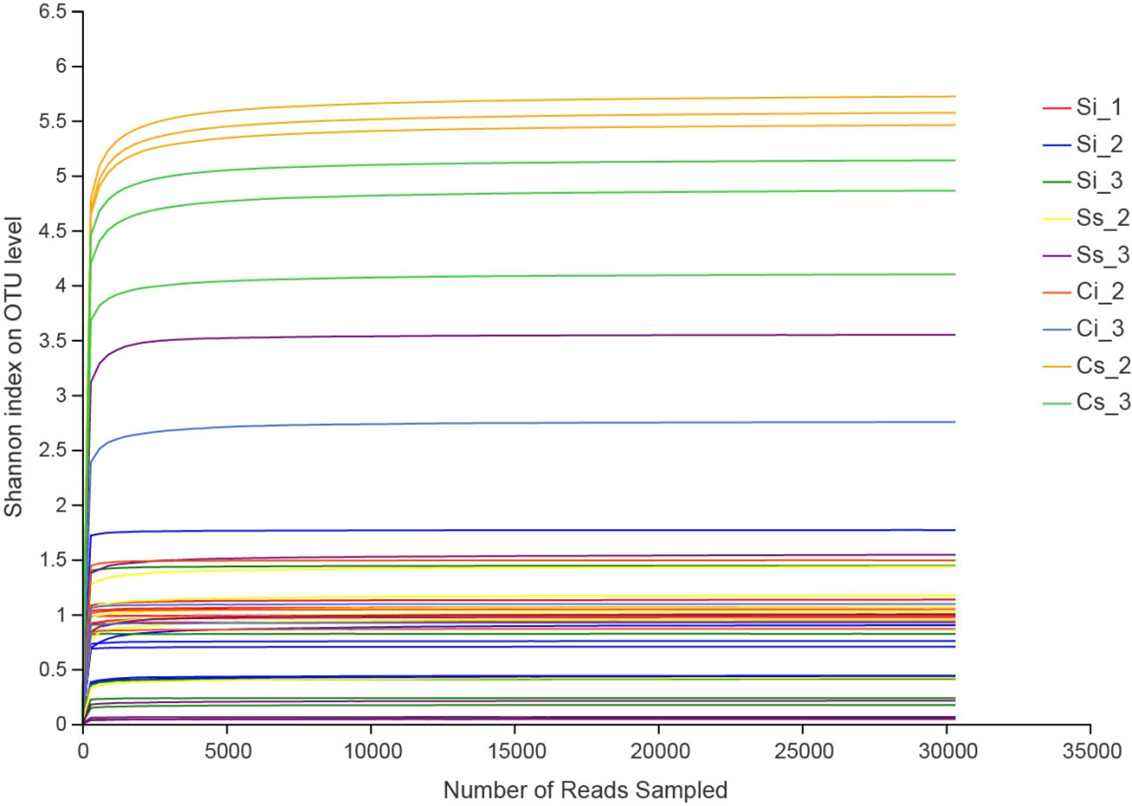


Fig. S1 Shannon-Winner index curve


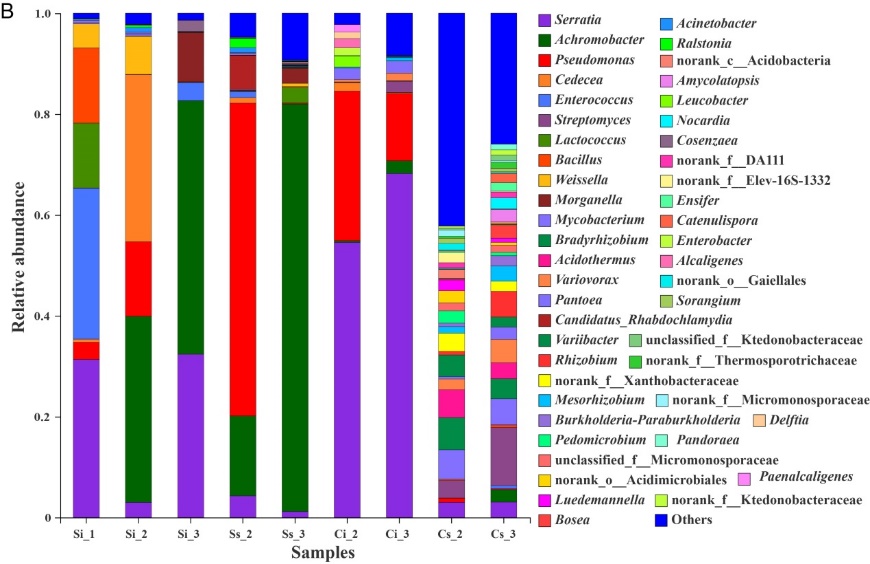


Fig.S2 Relative abundance histogram of bacterial community composition. A: Community composition at Phylum level. B: Community composition at the Genus level.
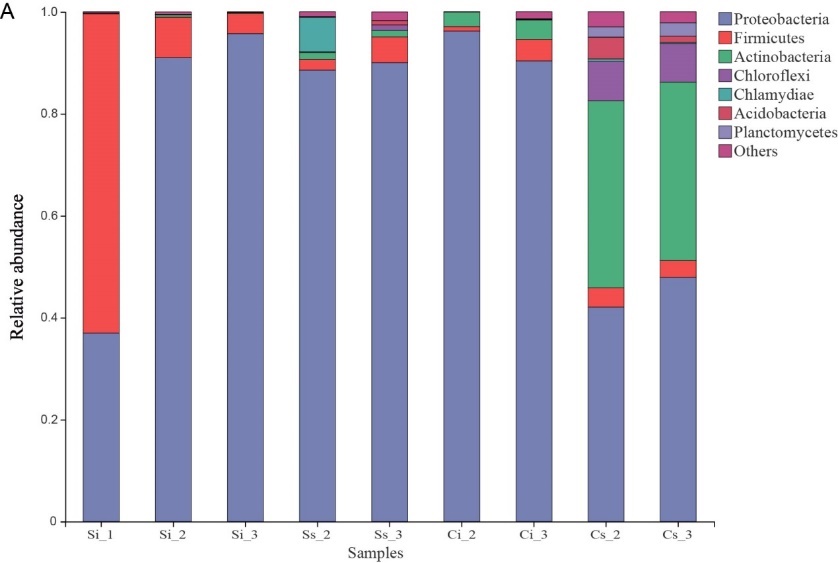


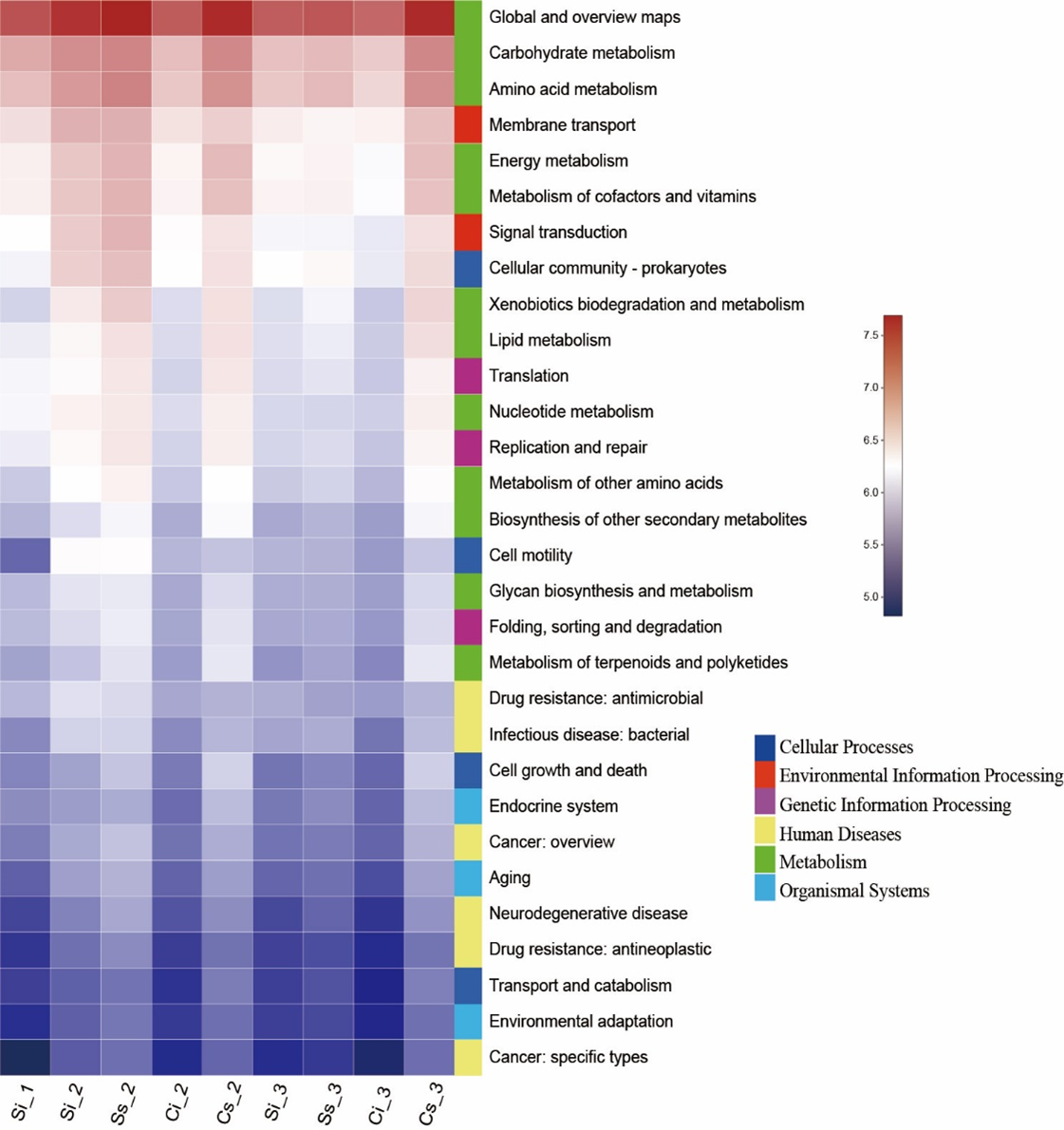


Fig. S3 Abundance heat map of bacteria KEGG pathway 2 in different samples with OTU levels


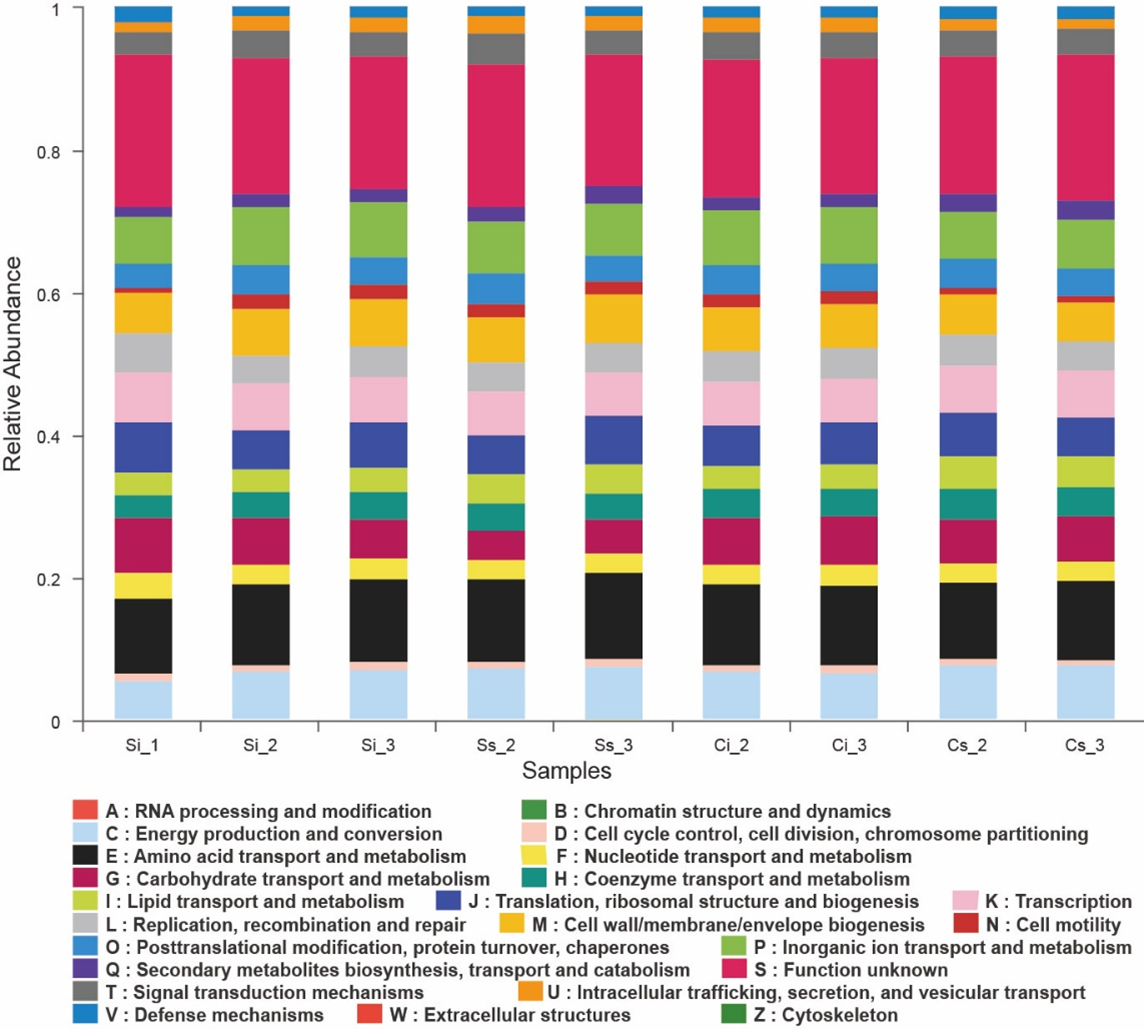


Fig. S4 Bacterial COG function classification of each sample at OTU level


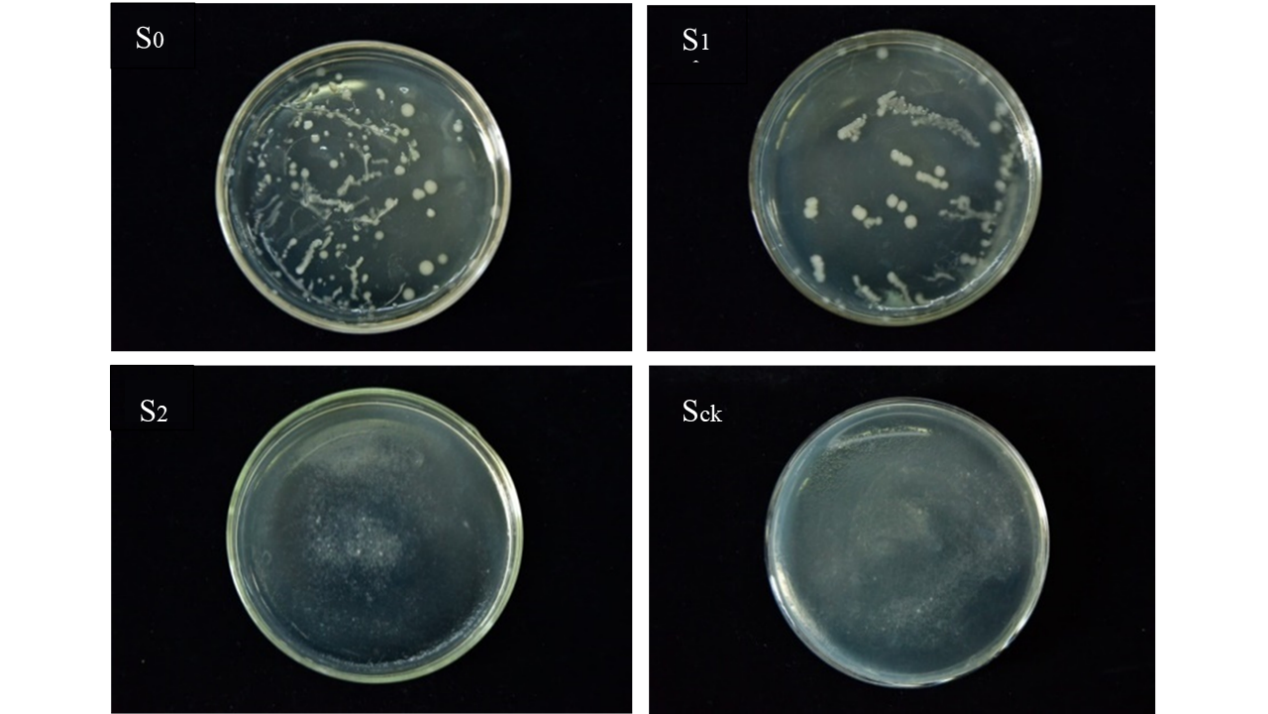


Fig. S5 The growth of single colonies of bacteria on plates in different treatment groups. S0: 10-7 concentration of soil liquid; S1: 600 mesh nylon mesh filtered soil suspension; S2: Soil suspension filtered by the fungus mycelium cortices of *Cordyceps chanhua*; Sck: Sterile water filtrate filtered by the mycelium cortices of *Cordyceps chanhua*.

Table S1 Atom% (15N) and N (total nitrogen) content in different samples of experiment E

| Samples | ^15^N (Sam) atom% | TN% |
| --- | --- | --- |
| E1 | 0.3758±0.0006a | 10.1633±0.5065a |
| E2 | 0.3782±0.0011a | 8.1233±0.8892b |
| E3 | 0.3780±0.0017a | 11.1000±0.5145c |
| E4 | 0.3711±0.0008b | 0.2433±0.0322d |
| Eck | 0.3716±0.0024b | 0.1967±0.0208d |

Note: E1: L-glutamic -^15^N+ conidia suspension injected silkworm pupa; E2: *C. chanhua* mycoderm; E3: Internal sclerotia sample; E4: Soil within 2cm of the *C. chanhua*; Eck: No injection of *C. chanhua* body Soil within 2cm.

Table S2 The content of atom% (15N) and N (total nitrogen)in different samples of experiment D

| Samples | ^15^N (Sam) atom% | N（总氮）% |
| --- | --- | --- |
| D1 | 0.4003±0.0096a | 0.2533±0.0513a |
| D2 | 0.3775±0.0072b | 7.1833±1.7420b |
| D3 | 0.3663±0.0005c | 10.3300±1.3455c |
| D4 | 0.3765±0.0060bc | 0.7700±0.4521a |
| Dck | 0.3659±0.0007c | 13.3433±0.3408d |

Note: D1: L-glutamic -^15^N treated labeled soil; D2: *C. chanhua* mycoderm; D3: Internal sclerotia sample; D4: Soil within 2 cm of the *C. chanhua*; Dck: The body of the *C. chanhua* not injected.


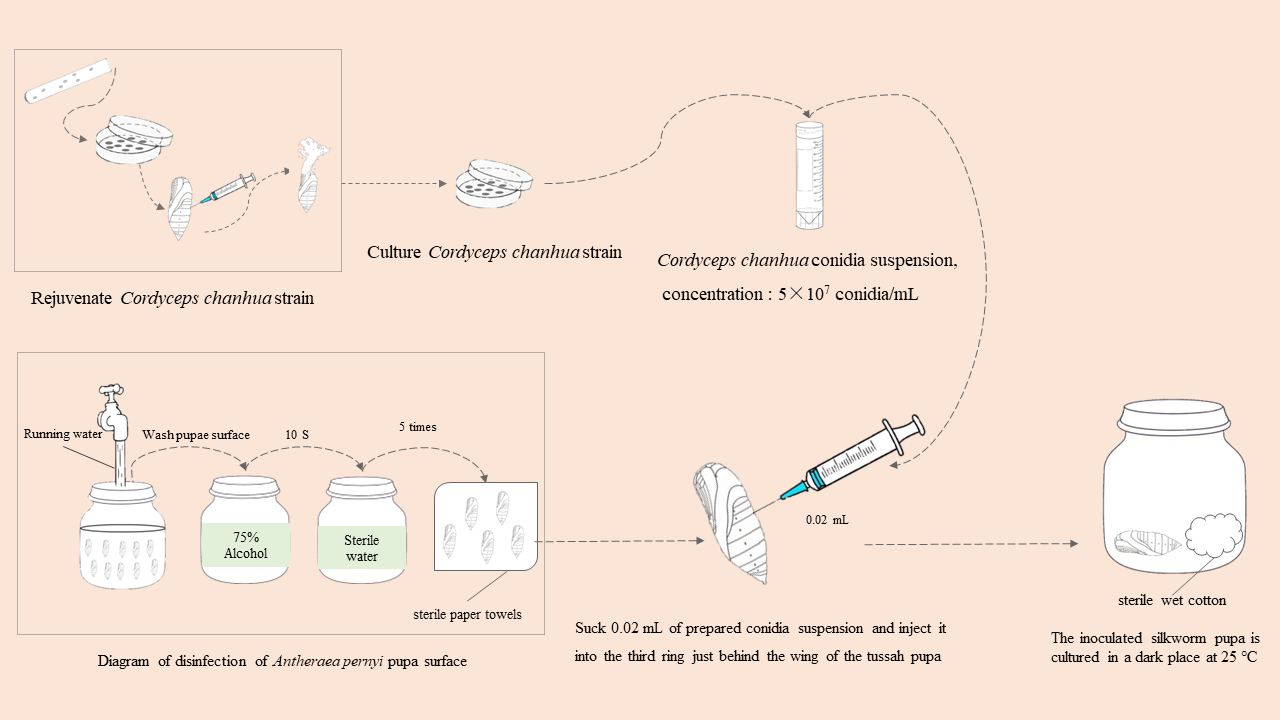


Fig. S6 *Cordyceps chanhua* cultivation process diagram.

**Table S3 Scanning electron microscopy to observe the structure and arrangement of the mycelium of *Cordyceps chanhua***

| Steps | Detailed methods |
| --- | --- |
| Immobilization | The sampling site should be determined with the fresh membrane tissue, and the mechanical damage such as stretching, contusion and extrusion should be minimized. The sample should be sampled within 1 to 3 min, and the tissue block area should not exceed 3 mm^2^. The sample surface should be gently rinsed with PBS, and the surface to be scanned should be identified and protected and marked. The fixation solution was quickly put into the electron microscope and fixed at room temperature for 2 h, and then transferred to 4 ℃ for preservation. |
| After- immobilization | The fixed sample was rinsed with 0.1 M phosphate buffer PB (pH 7.4) for 3 times, 15 min each time. Use 0.1 M phosphate buffer PB (pH 7.4) to prepare 1% osmic acid and fix it at room temperature and away from light for 1-2 h. After fixation, rinse with 0.1M phosphate buffer PB (pH 7.4) for 3 times, 15 min each time. |
| Dehydration | The immobilizated samples were injected with 30%, 50%, 70%, 80%, 90%, 95% and 100% alcohol for 15 min each time, and then isoamyl acetate for 15 min for dehydration. |
| Dry and conductive treatment | Put the sample into the critical point dryer for drying, and then put the sample closely on the conductive carbon film double-sided tape into the ion sputtering instrument sample platform for spraying gold for about 30s. |
| Observation and picture saving | Scanning electron microscope was used to observe the image area, and the images were collected and saved at the scales of 10 μm and 5 μm respectively. |
| Density calculation | Image-Pro Plus 6.0 software was used to count the number of mycelia in each section with a 3.0 k 10 μm scale as the standard. The number of hypha was divided by the section area to obtain the hypha density of the mycelium membrane. |


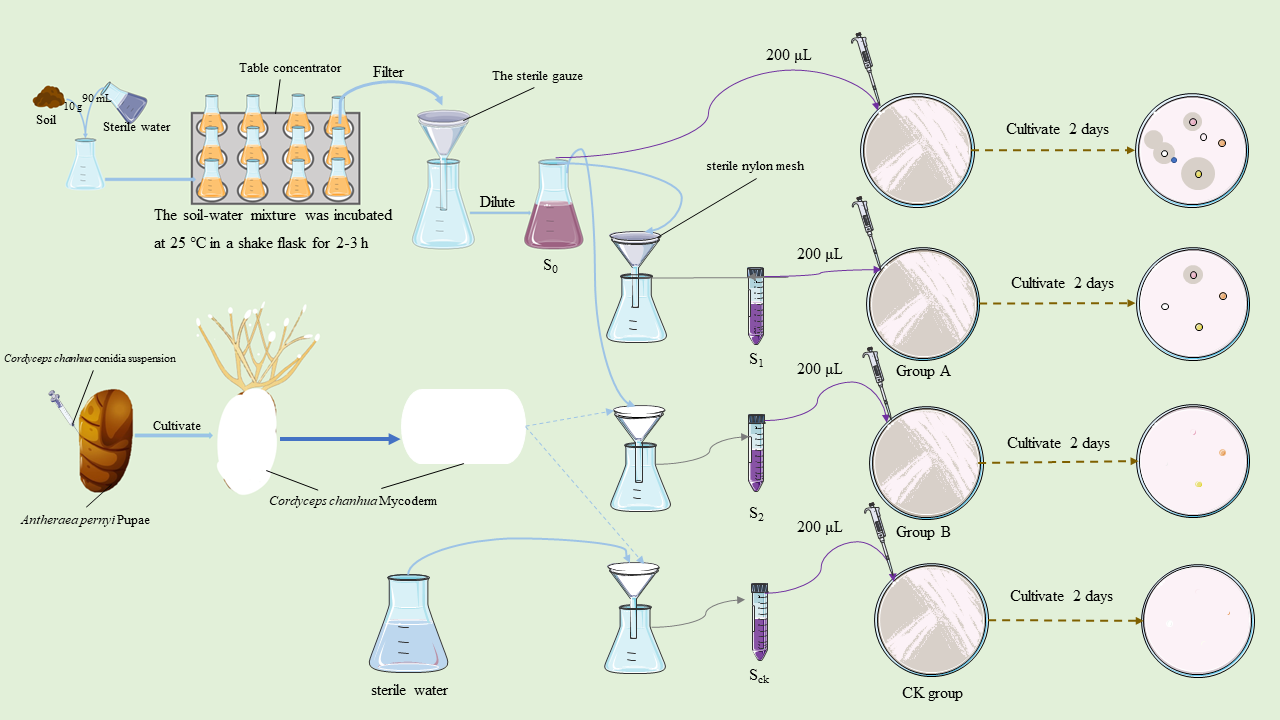


Fig. S7 Schematic diagram of experimental process and sampling of the filtration effect of *Cordyceps chanhua* mycoderm on bacteria.


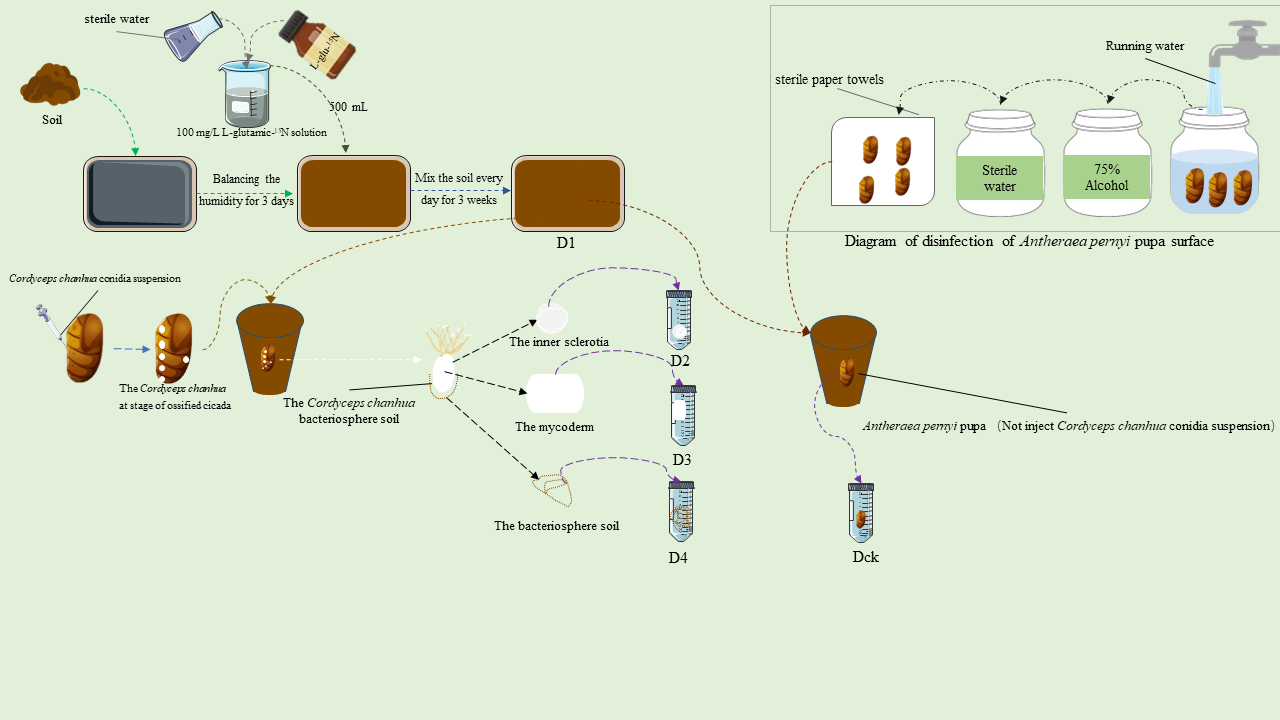


Fig. S8 Schematic diagram of experimental process and sampling of ^15^N flowing from soil to insect body through *Cordyceps chanhua* mycoderm.


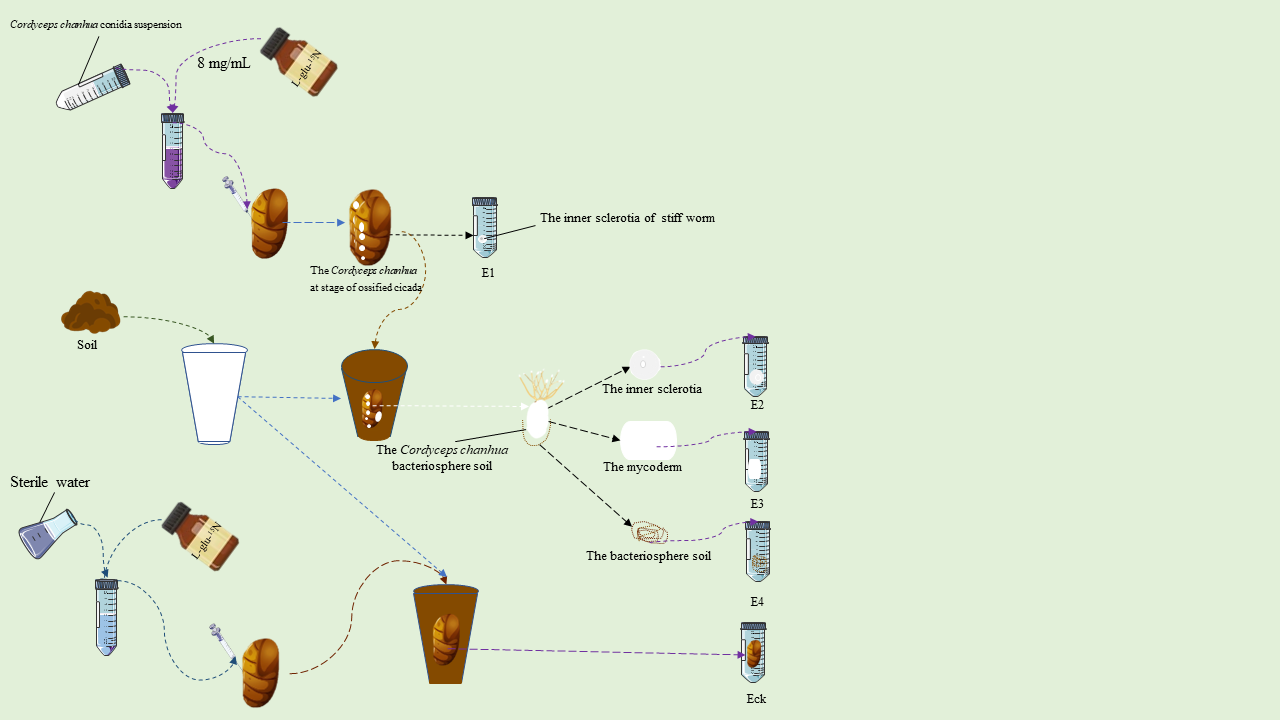


Fig. S9 Schematic diagram of experimental process and sampling of 15N flowing from insect body to soil through *Cordyceps chanhua* mycoderm.
